# Progesterone in women with arrested premature labor, a report of a randomised clinical trial and updated meta-analysis

**DOI:** 10.1186/s12884-017-1400-y

**Published:** 2017-08-02

**Authors:** Stephen Wood, Yacov Rabi, Selphee Tang, Rollin Brant, Susan Ross

**Affiliations:** 1 Department of Obstetrics and Gynaecology, University of Calgary, 4th Floor, North Tower, Foothills Medical Centre 1441 - 29th Street NW, Calgary, AB T2N 2T9 Canada; 2 Alberta Children’s Hospital Research Institute, Foothills Medical Centre, Room, rm C211 1403 - 29th Street NW, Calgary, AB T2N 2T9 Canada; 3 Department of Statistics, University of British Columbia, ESB rm 3146, 2207 Main Mall F512-4480 Oak Street, Vancouver, BC Canada; 4 Obstetrics & Gynecology, University of Alberta, 5S141 Lois Hole Hosp/Robbins Pav/RAH, Edmonton, AB T5H 3V9 Canada

**Keywords:** Premature labor, Maintenance tocolysis, Progesterone, Clinical trial, Meta-analysis

## Abstract

**Background:**

Progesterone may be effective in prevention of premature birth in some high risk populations. Women with arrested premature labor are at risk of recurrent labor and maintenance therapy with standard tocolytics has not been successful.

**Methods:**

Randomized double blinded clinical trial of daily treatment with 200 mg vaginal progesterone in women with arrested premature labor and an updated meta-analysis.

**Results:**

The clinical trial was terminated early after 41 women were enrolled. Vaginal progesterone treatment did not change the median gestational age at delivery: 36+2 weeks versus 36+4 weeks, *p* = .865 nor increase the mean latency to delivery: 44.5 days versus 46.6 days, *p* = .841. In the updated meta-analysis, progesterone treatment did reduce delivery <37 weeks gestation and increase latency to delivery, but this treatment effect was not evident in the high quality trials: (OR 1.23, 95% CI 0.91, 1.67) and (−0.95 days, 95% CI −5.54, 3.64) respectively.

**Conclusion:**

Progesterone is not effective for preventing preterm birth following arrested preterm labor.

## Background

A significant number of women who eventually deliver prematurely present with threatened preterm labor. Despite the success of initial tocolysis [1], many will develop recurrent labor and go on to deliver prematurely. These patients represent an opportunity for the secondary prevention of prematurity. Unfortunately, drugs such as calcium channel blockers [2], non-steroidal anti-inflammatories [3] and antosiban [4] have not been clearly effective in maintenance tocolysis. Based on the success of progesterone treatment in preventing prematurity in some high risk populations [5, 6], we initiated a clinical trial of progesterone in women with arrested premature labor. We report the results of our trial of treatment with 200 mg vaginal progesterone in women with arrested premature labor along with a meta-analysis of all types of progesterone for maintenance tocolysis.

## Methods

### Randomized Clinical Trial

Women presenting in premature labor to our center, the Foothills Medical Center in Calgary, Alberta, Canada, were approached by the primary investigator or study nurse for enrollment in the study. Inclusion criteria were women with gestational age 23^0^–32^6^ weeks with symptomatic contractions successfully arrested for at least 12 h with tocolytics or those with contractions that spontaneously resolved but had a positive vaginal fetal fibronectin (>50 ng/ml). Exclusion criteria were multiple pregnancy, placenta previa, preterm premature rupture of membranes (PPROM) at presentation and any contraindication to progesterone use. Consenting subjects were allocated by a randomization schedule developed by the trial statistician (R.B) using a random number generator and in random blocks of 2 or 4. Randomization was stratified into two strata to ensure balance in these important risk factors for preterm birth between the two groups: (i) tocolytic use; (ii) no tocolytic use. The primary investigators and study personnel involved in recruitment were not aware of the allocation sequence. Treatment packs containing 200 mg tablets of either micronized progesterone (Utrogestan, Besins-Healthcare) or an identical placebo were dispensed by a central research pharmacy. The treatment duration was from the time of randomization until 35^6^ weeks gestation or until delivery of the fetus, if sooner. The primary outcomes of the trial were gestational age at delivery and latency to delivery. Outcome assessors were blind to treatment status. The expected date of delivery recorded in the chart, at the time of randomization, was used subsequently to determine gestational age at delivery. Secondary outcomes included delivery <37 and <35 weeks gestation, recurrent premature labor and neonatal outcomes including death, broncho-pulmonary dysplasia, intraventricular hemorrhage, necrotizing enterocolitis, respiratory distress syndrome, hyperbilirubinemia, sepsis and need for ventilation. Our planned sample size was 60 per group, based on a 90% power to detect a 2 week difference in gestational age at delivery [7] at a significance level of (0.05). The outcomes were analyzed by intention to treat with the subjects remaining in the group they were randomized to, regardless of compliance with treatment. The gestational age at delivery were compared between the two groups using the Mann-Whitney *U* test, as the data was skewed. Mean latency to delivery was assessed using the student’s *t*-test. For preterm delivery <37 weeks and <35 weeks, a relative risk was calculated and statistical significance assessed by Fisher’s exact test. The other secondary outcomes were analyzed similarly. The trial was registered at ClinicalTrials.gov (NCT01286246). The trial was reported following CONSORT guidelines.

### Meta-analysis

The primary research question of the meta-analysis was: Does maintenance tocolysis with progesterone prevent prematurity, (<37 and <34 weeks gestation) or extend latency to delivery? The secondary question was: Does treatment reduce perinatal mortality. A literature search up to April 2015 was performed using the following databases and MeSH terms: Medline (Tocolytic Agents, or Tocolysis, or Obstetric labor, premature and Progesterone and Clinical trial), Embase (Premature labor or Tocolysis and Progesterone and Clinical trial), PubMed (premature labor and Progesterone and Clinical trial) and the Cochrane Central Register of Controlled Trials (Obstetric labor, premature or Tocolysis and Progesterone). These searches were subsequently updated in October 2015 and again in April 2016 and February 2017. The identified abstracts and appropriate manuscripts were reviewed by two of the authors (SW, SR). Studies were included if they were clinical trials of progesterone in women with premature labor following tocolysis. Risk of bias and quality of the manuscripts was judged independently by the reviewers using standard criteria [8]. All studies were graded as high or low quality based on four key quality indicators: adequate randomization and allocation concealment, blinding, limited losses to follow up (<20%) and intention to treat analysis. Studies that were deficient in any of these areas were graded as low quality. Studies were not included in the quantitative summary analysis unless intent to treat analysis was presented or could be calculated from the available data. Authors were contacted to obtain missing data. The primary outcomes of the meta-analysis were premature delivery <37 and <34 weeks gestation and latency to delivery. Quantitative analysis with a fixed and random effects models were performed with RevMan 5.1. Statistical assessment for heterogeneity was performed and considered statistically significant if *p* < .05. Random effects models were used for analyses with significant heterogeneity. A subgroup analysis by type of progesterone (oral, vaginal or intra-muscular) and by trial quality was planned a priori. This analysis was done with a fixed effects model even if there was significant heterogeneity between high and low quality studies then pooled analysis of all studies would not be meaningful. The meta-analysis results were reported as per PRISMA guidelines.

## Results

### Randomized Clinical Trial

Between February 2011 and February 2014 41 women were enrolled in the trial. Unfortunately, this did not meet our recruitment goals. Furthermore, the study drug we had been provided reached an expiry in August 2014 and the provider had changed its progesterone product. To continue the trial a new application would have been necessary to Health Canada. Given all these factors a decision was made to terminate the trial.

The patient characteristics are provided in the Appendix Table 3 and were adequately balanced between the two groups. The flow diagram for the trial is provided in Appendix Figure 8. There were no losses to follow up nor post randomization exclusions. Treatment with progesterone, compared to placebo, did not result in an increase in median gestational age at delivery: 36^+2^ weeks versus 36^+4^ weeks, mean difference − .0^2^ (−3^+1^ to 2^+3^) nor in mean latency to delivery: 44.5 days versus 46.6 days, mean difference − 2.1 (−22.8, 16.8). No significant differences were detected in any of the secondary outcomes, Appendix Table 4. There were no perinatal deaths. Only 50% of subjects returned their treatment diaries and unused capsules so reliable estimates of compliance could not be made.

### Meta-analysis

**Fig. 1:**
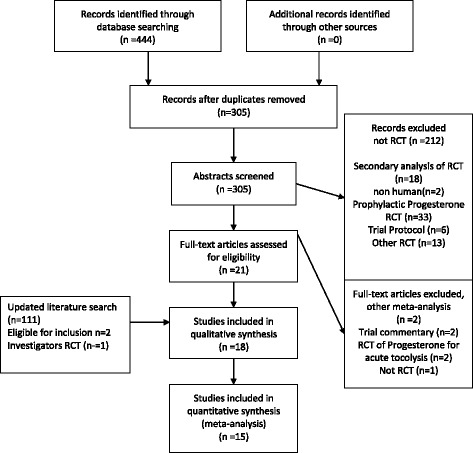
Flow diagram of studies included in the meta-analysis

**Fig. 2:**
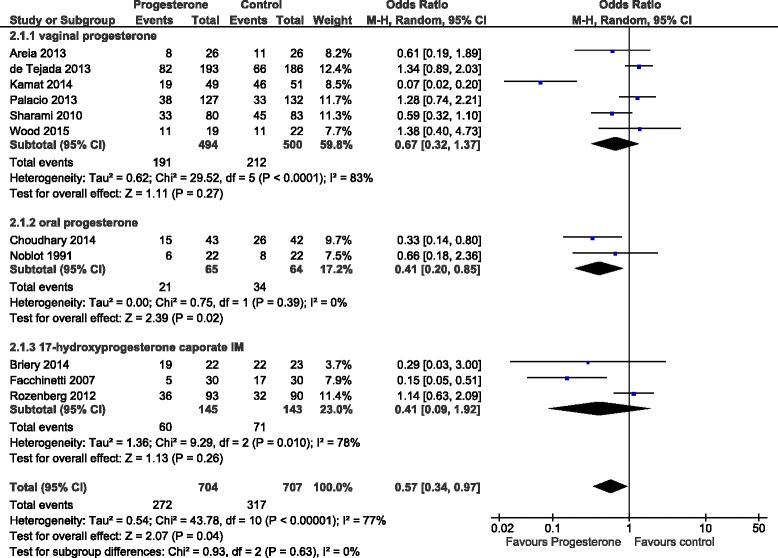
Forest plot for the outcome: Delivery less than 37 weeks comparing treatment with progesterone to controls

**Fig. 3:**
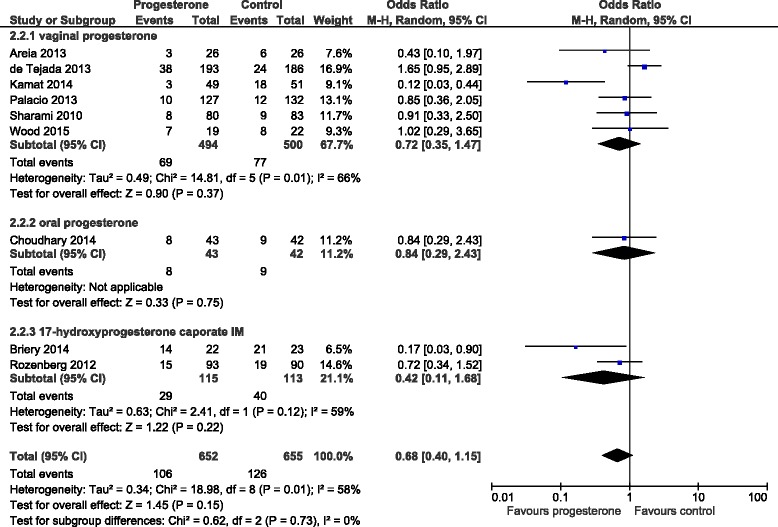
Forest plot for the outcome: Delivery less than 34 weeks comparing treatment with progesterone to controls

**Fig. 4:**
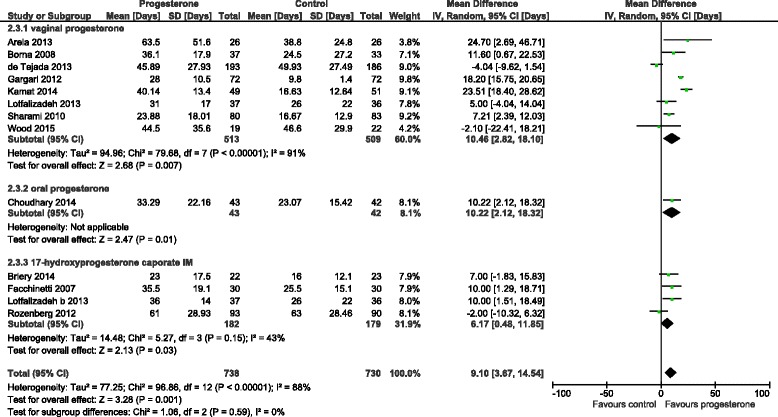
Forest plot for the outcome latency to delivery (days) comparing treatment with progesterone to controls

**Fig. 5:**
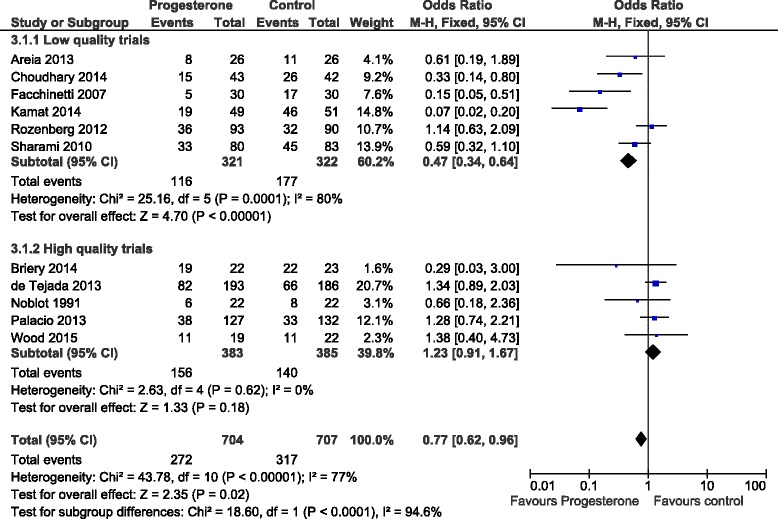
Forest plot for the outcome: Delivery less than 37 weeks comparing treatment with progesterone to controls stratified by trial quality

**Fig. 6:**
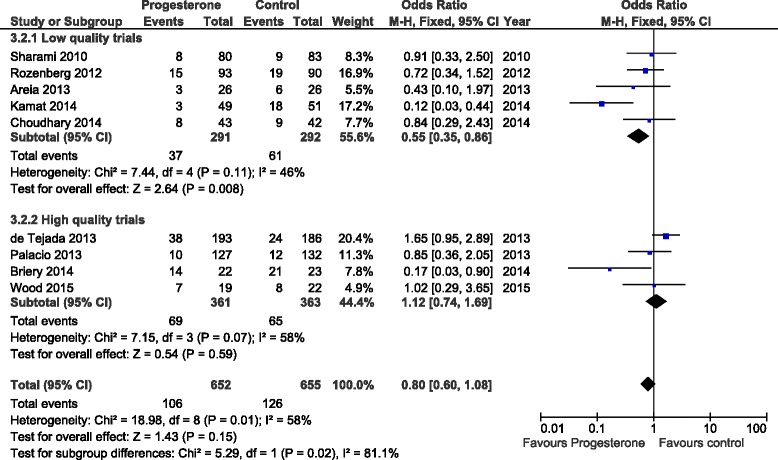
Forest plot for the outcome: Delivery less than 34 weeks comparing treatment with progesterone to controls, stratified by trial quality

**Fig. 7:**
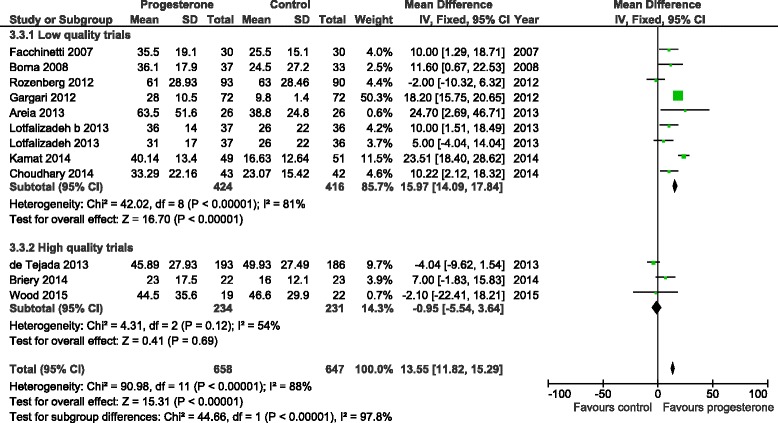
Forest plot for the outcome latency to delivery (days) comparing treatment with progesterone to controls, stratified by trial quality

The initial literature search initially identified 96 (Medline), 167 (Embase), 163 (PubMed) and 18 (Cochrane) abstracts. The updated literature search identified a further 60 abstracts. The details of the review process are presented in Fig. 1. Ultimately, 18 trials (including ours) were identified and 15 were included in the meta-analysis. The reasons for exclusion were: two trials were single dose progesterone treatment for acute tocolysis only not maintenance tocolysis [9, 10] and one was a trial of prophylactic 17-hydroxyprogesterone caporate in women with previous premature delivery [11]. The details of the included trials are presented in Table 1. Two trials employed oral progesterone [12, 13], three used intra-muscular 17-hydroxyprogesterone caporate [14–16], our trial and seven others used vaginal progesterone [17–23] and one trial compared intramuscular 17-hydroxyprogesterone caporate or vaginal progesterone to no treatment [24]. Eight authors were contacted by email requesting additional information regarding outcomes not reported in their manuscripts and responses were received from three. All the trials were reviewed for quality by two reviewers (SW, JR). Five were judged to be high quality and nine low quality (Table 2). Overall, treatment with progesterone reduced preterm birth less than 37 weeks gestation (OR 0.77, 95% CI 0.62, 0.96). However, this was not statistically significant for the vaginal progesterone nor intra-muscular 17-hydroxyprogesterone caporate subgroups (Fig. 2). The proportion of births before 34 weeks gestation was also reduced with treatment (OR 0.80, 95% CI 0.60, 1.08) but this difference was not statistically significant (Fig. 3). Additionally, a statistically significant increase in latency to delivery was apparent for all three progesterone treatments (Fig. 4). Planned subgroup analysis by quality revealed that the results varied significantly between high and low quality trials (Figs. 5, 6 and 7). The pooled results of the low quality trials suggested a reduction in the risk of delivery less than 37 weeks (OR 0.47, 95% CI 0.34, 0.64), less than 34 weeks (OR 0.55, 95% CI 0.35, 0.86) and increased latency to delivery (15.97 days, 95% CI 14.09, 17.84). However, the summary of high quality trials did not reveal any benefit for any of the outcomes: delivery less than 37 weeks (OR 1.23, 95% CI 0.91, 1.67), delivery less than 34 weeks (OR 1.12, 95% CI 0.74, 1.69) nor latency to delivery (−0.95 days, 95% CI −5.54, 3.64). Only five trials reported any perinatal deaths, 31 in the low quality trials and 9 in the high quality trials. Progesterone treatment was associated with a reduction in risk of perinatal death in the low quality trials: (OR 0.39, 95% CI 0.17, 0.87) but not the high quality trials: (OR 0.52, 95% CI 0.14, 1.95).

**Table 1:** Study characteristics of randomized controlled trials of progesterone for maintenance tocolysis

Study	Year	Country	Number of Subjects	Study Population	Treatment/controls
Areia [22]	2013	Portugal	52	24–34 weeks gestation cervical length ≤ 25 mm after tocolysis with antosiban.	Progesterone 200 mg vaginally/No treatment.
Borna [21]	2008	Iran	70	24–34 weeks gestation after tocolysis with MgSO4, cervical dilation ≤2 cm.	Progesterone 400 mg vaginally/No treatment.
Choudhary [13]	2014	India	90	24–34 weeks gestation after tocolysis with nifedipine, cervical dilation >1 cm.	Progesterone 200 mg orally/Placebo
Facchinetti [15]	2007	Italy	60	25–34 weeks gestation after tocolysis with antosiban.	17-hydroxyprogesterone caporate 341 mg IM q 4 days/ No treatment
Kamat [20]	2014	India	110	<37 weeks gestation, after tocolysis with nifedipine.	Progesterone 400 mg vaginally/nifedipine 20 mg q8h.
Lotfalizadeh [24]	2013	Iran	110	26–36 weeks gestation, after tocolysis with nifedipine or MgSO4	Progesterone 400 mg vaginally, or 17-hydroxyprogesterone caporate 250 mg IM/No treatment.
Noblot [12]	1991	France	44	<37 weeks gestation, regular contractions, tocolysis with ritodrine	Progesterone 400 mg orally, started before cessation of contractions/Placebo.
Palacio [19]	2013	Spain	265	24–34 weeks gestation, cervical length ≤ 25 mm. Arrested preterm labour, tocolytic not specified	Progesterone 200 mg vaginally/Placebo.
Rozenberg [14]	2012	France	188	24–31 weeks gestation and cervical length < 25 mm after tocolysis	17-hydroxyprogesterone caporate 500 mg biweekly/No treatment.
Sharami [18]	2010	Iran	173	28–36 weeks gestation, tocolysis with MgS04	Progesterone 200 mg vaginally/Placebo.
de Tejada	2015	Switzerland and Argentina	384	24–33 weeks gestation, tocolysis with β-mimetics, antosiban, or calcium channel blockers.	Progesterone 200 mg vaginally/Placebo.
Briery [16]	2014	USA	45	20–30 weeks gestation, tocolysis with NSAIDS, nifedipine or MgSO4	17-hydroxyprogesterone caporate 250 mg IM weekly/ Placebo
Gargari [23]	2012	Iran	110	24–33 weeks gestation, tocolysis with MgSO4	Progesterone 400 mg vaginally/No treatment
Wood	2017	Canada	41	23–33 weeks gestation, after tocolysis with NSAIDS or nifedipine or no tocolysis and positive vaginal fetal fibronectin	Progesterone 200 mg vaginally/Placebo.

**Table 2:** Quality assessment of randomized controlled trials of progesterone for maintenance tocolysis

Study	Randomization/Allocation concealment method	Blinding	Compliance with treatment	Post Randomization Exclusions	Intention to Treat analysis	Quality	Included in meta-analysis
Areia	“no allocation concealment”	No	None discontinued treatment	No	Yes	Low	Yes
Borna	Random number list, no allocation concealment	No	Unclear	No	Yes	Low	Yes
Choudhary	Computer generated list, “randomly allocated by third party”	Yes	Unclear	Yes, 2 in progesterone group, 3 in placebo group	Yes	Low	Yes
Facchinetti	Computer generated list, “managed by the senior midwife”	No	“Patients were compliant”	No	Yes	Low	Yes
Kamat	Computer generated random number table. Unclear allocation concealment	No	Unclear	Yes, 4 in progesterone group 6 in control.	Yes	Low	Yes
Lotfalizadeh	No information provided.	No	Unclear	?	?	Low	Yes
Noblot	Randomized schedule prepared by pharmacy	Yes	Unclear	None	Yes	High	Yes
Palacio	Centralized computer randomization	Yes	Unclear	Yes 6 in progesterone group.	Yes	High	Yes
Rozenberg	Centralized computer randomization.	No		Yes, 1 in progesterone group and 4 in control.	Yes	Low	Yes
Sharami	“randomized into two groups using the random block allocation method”	Yes	Unclear	Yes, 6 in progesterone group, 4 in placebo	Yes	Low	Yes
de Tejada	Centralized computer randomization.	Yes	4 in progesterone and 5 in placebo groups stopped medication. Overall 58% compliance	Yes 4 in progesterone group, 2 in placebo.	Yes	High	Yes
Briery	Sequentially numbered sealed opaque envelopes	Yes	Unclear	None	Yes	High	Yes
Gargari	Unclear	No	Unclear	Yes, 38 subjects	?	Low	Yes
Wood	Pharmacy randomization with schedule concealed from clinicians.	Yes	Unclear	None	Yes	High	Yes

## Discussion

Neither our trial result nor the meta-analysis suggest progesterone is effective for maintenance tocolysis. Although the overall pooled analysis in the meta-analysis favors a treatment effect, the high quality studies do not. One previously published meta-analysis of vaginal progesterone for maintenance tocolysis concluded that treatment was associated with a reduced risk of prematurity and prolongation of pregnancy [25]. However, no subgroup analysis by study quality was reported. Overall, we feel that the results of the high quality studies are the most reliable indicator of a null treatment effect. If anything, it could be argued that these studies suggest a potential for harm with as the point estimates suggested an increase in prematurity and shorter latency with treatment. The substantial effect of trial quality that we observed has been reported previously. Several groups have found that poor allocation concealment and blinding influence results, usually, by increasing positive treatment effects [26–28]. Although some readers may prefer the results of the pooled analysis of all the trials, we would caution against this. The history of clinical trials has taught trialists the importance of protecting their research from their own biases by using good study design. Therefore, results from studies with good design and execution are more likely to produces reliable estimates of effect. In this instance, such studies do not suggest progesterone, by any route, is an effective therapy for prolonging pregnancy in women who have arrested premature labor.

Still, alternative explanations should be considered for the negative results of both our trial and the meta-analysis. One typical issue, low power, is not one we feel is likely. Although latency to delivery is not necessarily, by itself, a clinically important endpoint, it should be a sensitive indicator of biologic effect. That we observe no discernible effect on latency in our trial and the summary of the high quality trials support a conclusion that progesterone is ineffective. It does remain a possibility that an effective progesterone dose has yet to be determined and future trials of increased doses may show positive results. Unfortunately, although our review found a fair number of studies that used higher doses of both vaginal and intra-muscular progesterone, none of these was high quality trial. A search of ClinicalTrials.gov only identified one additional, terminated, and unreported trial that used 400 mg vaginal progesterone which recruited 7 subjects (NCT00946088). Poor patient compliance is also a potential explanation for our results. Regrettably, in our study, we were unable to reliably comment on compliance, as too few subjects returned their pill diaries or unused medications. This did not seem to be unique to our trial as very few of the studies reported any measures of patient compliance. While in the case of treatment with intra-muscular 17-hydroxyprogesterone caporate, poor compliance may be evident to the investigators, the same cannot be said of vaginal or oral routes.

## Conclusions

In summary, our results do not support the routine use of progesterone, in any form, for maintenance tocolysis. However, further clinical trials with good measurement of patient compliance and higher doses of progesterone could be justified.

## Figures and Tables

**Fig. 8:**
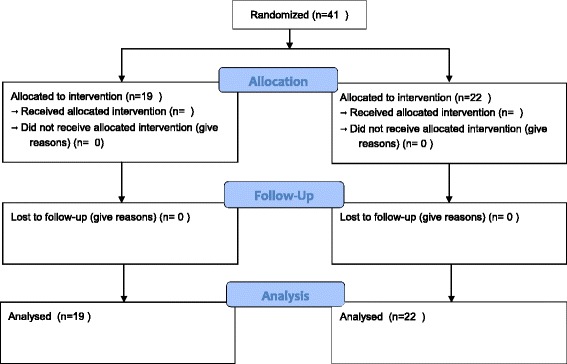
Consort Diagram

## Appendix

**Table 3:** Patient Characteristics

Characteristic	Progesterone *n* = 19	Placebo *n* = 22
Mean Maternal age (years)	26.5 (SD 3.9) Range 20–34	29.1 (SD 5.9) Range 19 to 39
Mean Gestational age at randomization (weeks and days)	29^+0^ (SD 2^+2^) Range 24^+2^ to 32^+5^	28^+4^ (SD 2^+5^) Range 23^+1^ to 32^+3^
Gravidity		
1	4 (21.1%)	7 (31.8%)
2	9 (47.4%)	7 (31.8%)
3 or more	6 (31.6%)	8 (36.4%)
Parity		
0	6 (31.6%)	10 (45.5%)
1	8 (42.1%)	7 (31.8%)
2 or more	5 (26.3%)	5 (22.7%)
Number of previous PTB <37 weeks		
0	10 (58.8%)	16 (72.7%)
1	5 (29.4%)	3 (13.6%)
2	2 (11.8%)	3 (13.6%)
Unknown	2	0
Conception (this pregnancy)		
Spontaneous	14 (93.3%)	17 (81.0%)
Ovulation induction alone	1 (6.7%)	3 (14.3%)
IVF	0 (0.0%)	1 (4.8%)
Unknown	4	1
Multiple reduction (before randomization)		
No	16 (100%)	22 (100%)
Unknown	3	0
Pregnancy complications (this pregnancy, before or at time of randomization)		
None	3 (17.6%)	10 (47.6%)
Diabetes	1 (5.9%)	0 (0.0%)
Uterine abnormality	0 (0.0%)	1 (4.8%)
Previous AP bleed	5 (29.4%)	3 (14.3%)
Recreational drugs	0 (0.0%)	1 (4.8%)
Smoker	6 (35.3%)	4 (19.0%)
Other^a^	7 (41.2%)	5 (23.8%)
Unknown	2	1

^a^ Other in Group A includes cardiac disease; crohns/ileostomy/mult bowel resections; epilepsy/fam hx von willebrands; hypothyroidism/hx post partum depression; and limb girdle muscular dystrophy/ marginal placenta previa/prev thyroidectomy. Other in Group B includes depression/prev ruptured spleen; hx genital herpes; migraines/small septate uterus; depression/maternal grade 2 cardiac murmer; asthmatic; small subchorionic bleed noted on 8w US; and two prev cs

**Table 4:** Outcomes to 28 days after delivery

	Progesterone *n* = 19	Placebo *n* = 22	*p*-value*	Median/Mean/Risk Difference (95% Confidence Interval)*
Primary outcome				
Median Gestational age at delivery (weeks and days)	36^+2^ (IQR 7^+4^) Range 26^+6^ to 41^+2^	36^+4^ (IQR 8^+0^) Range 24^+3^ to 41^+2^	0.865	−0^+2^ (−3^+1^ to 2^+3^)
Mean Latency from randomization to delivery (days)	44.5 (SD 35.6) Range 0 to 113	46.6 (SD 29.9) Range 2 to 116	0.841	−2.1 (−22.8 to 18.6)
Secondary outcomes				
Maternal outcomes				
Gestational age at delivery <37 weeks	11 (57.9%)	11 (50.0%)	0.758	7.9% (−23.1% to 38.0%)
Gestational age at delivery <35 weeks	8 (42.1%)	9 (40.9%)	>0.999	1.2% (−29.5% to 31.4%)
Gestational age at delivery <34 weeks	7 (36.8%)	8 (36.4%)	>0.999	0.5% (−30.0% to 30.4%)
Tocolytics at any time during this pregnancy				
Yes	14 (77.8%)	16 (84.2%)	0.693	−6.4% (−36.3% to 26.3%)
No	4 (22.2%)	3 (15.8%)		
Unknown	1	3		
PPROM				
Yes	3 (16.7%)	4 (18.2%)	>0.999	−1.5% (−32.0% to 28.8%)
No	15 (83.3%)	18 (81.8%)		
Unknown	1	0		
Betamethasone treatment				
Yes	15 (93.8%)	18 (94.7%)	>0.999	−1.0% (−33.1% to 31.6%)
No	1 (6.3%)	1 (5.3%)		
Unknown	3	3		
Mode of delivery				
Vaginal	9 (47.4%)	16 (72.7%)	0.493	14.1% (−16.5% to 43.2%)
Operative vaginal	3 (15.8%)	1 (4.6%)		
Cesarean Section	7 (36.8%)	5 (22.7%)		
Delivery complications				
None	15 (83.3%)	18 (90.0%)	0.653	6.7% (−24.7% to 38.1%)
Hypertension	0 (0.0%)	1 (5.0%)		
AP bleed	0 (0.0%)	1 (5.0%)		
Other^a^	3 (16.7%)	0 (0.0%)		
Unknown	1	2		
Infant outcomes				
Median Birth weight (grams)	2625 (IQR 1340) Range 750 to 3766 (missing = 1)	2660 (IQR 1555) Range 670 to 3955	0.734	−105 (−739 to 506)
Adverse outcomes			0.732	9.5% (−23.9% to 41.1%)
One or more	10 (66.7%)	12 (57.1%)		
None	5 (33.3%)	9 (42.9%)		
Unknown	4	1		
Description of outcomes (could be more than one)				
Stillbirth/neonatal death	0	0		
BPD	0	3		
IVH	0	0		
NEC	0	1		
RDS	4	7		
Apnea/bradycardia	1	2		
Jaundice/hyperbilirubinemia	4	6		
Suspected sepsis	2	2		
Transient tachypnea	1	2		
Other^b^	8	20		
On ventilator	0	4		
Median Baby length of stay (days)	6 (IQR 29) Range 0 to 86 (missing = 3)	3 (IQR 38) Range 1 to 127 (missing = 4)	0.903	0 (−15 to 10)

^a^ Other includes: 3rd degree tear, maternal pancreatitis, and assisted breech extraction

^b^ Other in Group A includes: ventriculomegaly, admission to nicu (reason unknown), hypoglycemia, ROP, pulmonary insufficiency, GERD, esophageal atrenia, bilateral echogenic kidneys, PDA, positive enterobacter CONS tracheal aspirate, edema, hyperkalemia, anemia, metabolic acidosis, acute renal insufficiency, nephrocalcinosis, HMD, bilateral inguinal hernia, SGA, RT hydro ureter. Other in Group B includes: hypoglycemia, small cleft palate, hypovolemia, query infection, hypotension, minor hypospadius, oral aversion, SGA

* Fisher’s exact test for categorical variables, Mann-Whitney U test or t-test for continuous variables; Risk difference and exact 95% confidence limits for categorical variables, Hodges-Lehmann estimation of location shift (median of differences) or difference in means and 95% confidence limits for continuous variables

## Abbreviations

CI: Confidence interval
OR: Odds ration
PPROM: Preterm premature rupture of membranes
